# Immune responses to tumor-unrelated antigens might predict adverse effects in patients treated with anti-PD-1 immunotherapy

**DOI:** 10.1038/s41423-022-00947-1

**Published:** 2022-11-15

**Authors:** Björn Nüse, Tim Holland, Jochen Mattner

**Affiliations:** 1 Mikrobiologisches Institut - Klinische Mikrobiologie, Immunologie und Hygiene, Universitätsklinikum Erlangen and Friedrich-Alexander-Universität (FAU) Erlangen-Nürnberg, Erlangen, Germany; 2 Medical Immunology Campus Erlangen, FAU Erlangen-Nürnberg, Erlangen, Germany

**Keywords:** Predictive markers, Cancer

The advent of checkpoint inhibition targeting programmed cell death protein 1 (PD-1) or its ligand (PD-L1) revolutionized cancer immunotherapy and significantly improved the treatment of different malignancies. However, not all immune cell subsets that express PD-1 also target tumor cells. Utilizing the immune response of the germinal center reaction to seasonal influenza vaccination as a readout, Herati et al. introduced vaccination as an analytical tool to predict adverse effects beyond anticancer responses [1].

**Fig. 1:**
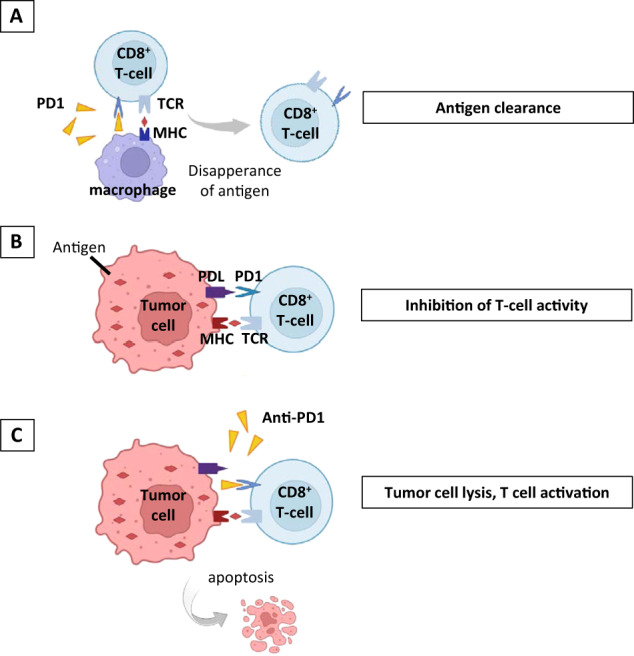
Influences of PD-1 expression and anti-PD-1 therapy on the cytotoxic CD8 T-cell (CTL) response. **A** Once the antigen is cleared, for example, in a tumor-free environment, PD-1 expression on CD8 + CTLs disappears. **B** Continuous TCR engagement by tumor antigens induces persistent PD-1 expression. Consequently, CTLs become exhausted and are no longer able to lyse tumor cells. **C** PD-1 checkpoint inhibition enables the reactivation of CTLs, leading to subsequent tumor cell lysis

Following rapid activation-induced upregulation, PD-1 (CD279) delivers inhibitory signals to B and T lymphocytes [2]. While PD-1 expression decreases again with antigen clearance (Fig. 1A), continued BCR or TCR ligation maintains PD-1 expression at high levels (Fig. 1B). Persistent PD-1 expression in chronic disease settings, such as cancer or viral infection, has been associated with a progressive loss of T-cell functions [3] (Fig. 1B). Conversely, blockade of PD-1 signaling redirects the activity of the immune system to treat malignancies, which is in part due to the recovery of exhausted CD8 T lymphocytes [4] (Fig. 1C). The discovery of PD-1 immune checkpoint blockade as a cancer therapy was even honored with the 2018 Nobel Prize in Physiology or Medicine.

**Fig. 2:**
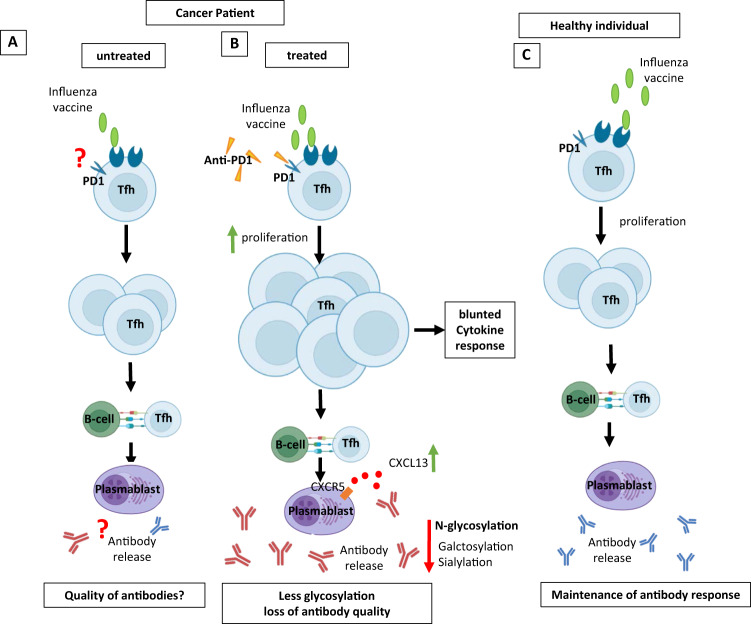
Qualitative and quantitative influences of anti-PD-1 checkpoint inhibition on follicular T-cell and humoral immune responses to a seasonal influenza vaccine. The Tfh-cell and plasmablast responses of cancer patients, either treated with therapies other than PD-1 (A) or with anti-PD-1 therapy B, and healthy individuals (C) are displayed. Anti-PD-1 therapy induced enhanced Tfh-cell proliferation and a more robust Tfh-cell–plasmablast germinal center reaction in only some anti-PD-1-treated patients

However, checkpoint inhibition targeting PD-1 also affects other PD-1-expressing B and T lymphocytes, including Tfh cells that do not respond to tumor antigens [5]. Thus, to investigate the role of PD-1 blockade in nontumor-related immune responses, Herati and colleagues compared Tfh- and B-cell responses to seasonal influenza vaccination among PD-1-treated and untreated cancer patients and healthy individuals (Fig. 2). Therefore, the authors enrolled adult patients with renal cell or urothelial carcinoma who were receiving anti-PD-1 immunotherapy in a first cohort. In a second patient cohort that they independently generated at a different institution, they enrolled adult patients with melanoma receiving immunotherapy and healthy individuals as controls. The participants received seasonal influenza vaccination on the same day as an infusion of anti-PD-1 immunotherapy. Blood was collected for different immunological analyses on the day of vaccination as a baseline, 1 week after vaccination and again three to six weeks after vaccination [1].

The authors first investigated the expansion of B cells and nonnaive CXCR5^+^ Tfh cells that simultaneously expressed ICOS and CD38 following PD-1 blockade. Compared to cancer patients who received therapies other than anti-PD-1 antibodies or healthy control individuals, patients with cancer receiving anti-PD-1 antibodies exhibited a 2.6-fold expansion of ICOS^+^CD38^+^ Tfh cells on average one week after vaccination (Fig. 2). However, only some patients exhibited this expansion of Tfh cells. Responses were transient and had returned to baseline levels at the late study time points. Although the frequency of plasmablasts was not greater in the anti-PD-1 group following vaccination, there was a subset of anti-PD-1-treated participants with robust induction of plasmablasts one week after vaccination. The plasmablast response correlated with the ICOS^+^CD38^+^ Tfh response, suggesting that anti-PD-1 therapy alters Tfh-cell–B-cell interactions. Accordingly, anti-PD-1-treated participants exhibited a substantial induction of plasma CXCL13, a biomarker of germinal center (GC) activity in secondary lymphoid tissues, one week after vaccination compared with baseline in both study cohorts (Fig. 2). Thus, the authors concluded that anti-PD-1 therapy presumably induces a robust enhancement of vaccine-induced cTfh-cell, plasmablast and GC activity.

Next, Herati et al. investigated whether anti-PD-1 therapy influences the induction of inhibitory antibodies against hemagglutinin (HAI), which serve as a correlate of protection following vaccination [6]. Consistent with a previous study [7], the application of PD1 checkpoint inhibition induced approximately 2-fold higher neutralizing antibody titers against all three strains of influenza included in the vaccine than no therapy. Moreover, similar to healthy adults, most anti-PD-1-treated adults were seroprotected. To assess the quality of the antibody response, the authors assessed the immunoglobulin (Ig) subclasses, glycosylation and affinity of the induced anti-hemagglutinin antibodies. Anti-PD-1-treated individuals exhibited alterations in the composition of IgG subclasses, showing dominant IgG1 responses, compared to controls. While afucosylation was not affected, the anti-hemagglutinin antibodies of anti-PD-1-treated patients exhibited reduced galactosylation and sialylation, two modifications that affect antibody function to confer protection against influenza in vivo [8]. Moreover, the baseline antibody affinity was lower in patients given anti-PD-1 therapy than in healthy controls. Thus, although anti-PD-1 therapy induces Tfh-cell, plasmablast and CXCL13 responses, it reduces the sialylation of anti-hemagglutinin antibodies at baseline and following vaccination, affecting the quality of influenza-specific antibodies (Fig. 2).

Given these quantitative and qualitative changes in antibody responses to the influenza vaccine, the authors also analyzed the composition of B-cell subsets. Anti-PD-1 therapy was associated with higher frequencies of plasmablasts even before vaccination. Induction of antibody release even without external triggers might unleash autoimmune complications [9]. The circulating plasmablast frequencies also correlated with the numbers of ICOS^+^CD38^+^ Tfh cells in anti-PD-1 patients before and after vaccination.

The transcriptional profiles of purified ICOS^+^CD38^+^ Tfh cells from anti-PD-1-treated adults revealed the upregulation of genes indicative of recent proliferation and downregulation of genes that mediate interferon signaling. Similarly, plasmablasts from vaccinated and anti-PD-1-treated patients upregulated genes consistent with greater proliferation relative to plasmablasts from healthy adults. The induction of these genes depended on vaccination. Anti-PD-1 treatment was also associated with the downregulation of IL-2/STAT5, IL6/JAK/STAT, IFN-γ and TGF-β signaling and apoptosis in activated Tfh cells. A second relevant transcriptional pattern of anti-PD-1 treatment was the downregulation of genes involved in the TNF/NFkB pathway, two signaling components regulating Tfh-cell biology [10]. Thus, anti-PD-1 therapy perpetuates the activation and proliferation of Tfh- and B-cell populations during the response to an influenza vaccine.

Finally, and most importantly, the authors investigated whether Tfh-cell biology offers insights into the mechanisms underlying adverse effects during anti-PD-1 therapy, a major limitation of current checkpoint blockade approaches. They observed higher expression of the ICOS protein and αIgG4, which they utilized as a proxy for PD-1 [4], in anti-PD-1-treated adults with adverse effects compared to anti-PD-1-treated adults who did not develop adverse effects. Transcriptional analyses confirmed 18 genes that were upregulated in activated Tfh cells from patients with adverse effects compared to those from patients without adverse effects. These included proliferation-associated genes and genes related to the cell cycle and cellular activation in patients with adverse effects. Thus, the activation and proliferation statuses as well as the blunting of cytokine pathway signaling in ICOS^+^CD38^+^ Tfh cells discriminate patients with adverse effects from patients without adverse effects following PD-1 checkpoint inhibition even before vaccination. As there were significant differences in the frequency of ICOS^+^CD38^+^ Tfh cells in patients with adverse effects after vaccination but not before vaccination, these data suggest that the Tfh-cell response following vaccination might help to distinguish patients with adverse effects from those without.

In summary, Herati and colleagues suggest Tfh-cell and plasmablast responses to influenza vaccination as potential valuable analytical tools to predict adverse effects in patients receiving anti-PD-1 therapy. Thus, immunoprofiling is pivotal to deciphering the key mechanisms underlying the altered immunological effects and responses in cancer immunotherapy patients. Thus, further studies need to define the regulation of PD-1 expression on the immune cells of cancer patients responding to the transient duration of these immune perturbations in larger cohorts once anti-PD-1 therapy has been discontinued.
